# Structural insights into the regulation of the human E2∼SUMO conjugate through analysis of its stable mimetic

**DOI:** 10.1016/j.jbc.2023.104870

**Published:** 2023-05-27

**Authors:** Stéphane Goffinont, Franck Coste, Pierre Prieu-Serandon, Lucija Mance, Virginie Gaudon, Norbert Garnier, Bertrand Castaing, Marcin Józef Suskiewicz

**Affiliations:** Centre de Biophysique Moléculaire (CBM), CNRS UPR, Orléans, France

**Keywords:** protein post-translational modifications (PTMs), ubiquitin-like modifications, SUMOylation, UBC9, SUMO, SUMO1, protein engineering, protein crystallography, protein oligomerisation

## Abstract

Protein SUMOylation is a ubiquitylation-like post-translational modification (PTM) that is synthesized through an enzymatic cascade involving an E1 (SAE1:SAE2), an E2 (UBC9), and various E3 enzymes. In the final step of this process, the small ubiquitin-like modifier (SUMO) is transferred from the UBC9∼SUMO thioester onto a lysine residue of a protein substrate. This reaction can be accelerated by an E3 ligase. As the UBC9∼SUMO thioester is chemically unstable, a stable mimetic is desirable for structural studies of UBC9∼SUMO alone and in complex with a substrate and/or an E3 ligase. Recently, a strategy for generating a mimetic of the yeast E2∼SUMO thioester by mutating alanine 129 of Ubc9 to a lysine has been reported. Here, we reproduce and further investigate this approach using the human SUMOylation system and characterize the resulting mimetic of human UBC9∼SUMO1. We show that substituting lysine for alanine 129, but not for other active-site UBC9 residues, results in a UBC9 variant that is efficiently auto-SUMOylated. The auto-modification is dependent on cysteine 93 of UBC9, suggesting that it proceeds *via* this residue, through the same pathway as that for SUMOylation of substrates. The process is also partially dependent on aspartate 127 of UBC9 and accelerated by high pH, highlighting the importance of the substrate lysine protonation state for efficient SUMOylation. Finally, we present the crystal structure of the UBC9–SUMO1 molecule, which reveals the mimetic in an open conformation and its polymerization *via* the noncovalent SUMO-binding site on UBC9. Similar interactions could regulate UBC9∼SUMO in some cellular contexts.

Protein SUMOylation is a eukaryotic protein post-translational modification (PTM) that plays an essential role in organisms from yeast to humans (reviewed in (1, 2, 3, 4)). It contributes to a variety of physiological cellular processes, including, among others, DNA replication, chromatin and transcription regulation, DNA repair, ribosome biogenesis, RNA splicing, nuclear trafficking, protein degradation, and cell cycle regulation. It also plays a role in disease (particularly cancer), therapy, and resistance.

At the molecular level, SUMOylation involves the covalent ligation of the small ubiquitin-like modifier (SUMO) to lysine residues on protein substrates (reviewed in (5, 6, 7)). The number of SUMO paralogues varies between organisms, with one (Smt3) in *Saccharomyces cerevisiae* and up to five in humans of which SUMO1, SUMO2, and SUMO3 are the best characterized and known to have partially distinct functions despite using the same core enzymatic cascade (8). Mature human SUMO2 and SUMO3 have nearly identical sequences (98%), while SUMO1 is distinct (47% identical to SUMO2/3). SUMO ligation depends on the successive activities of a single SUMO E1 activating enzyme (the SAE1:SAE2 heterodimer), a single SUMO E2 conjugating enzyme (UBC9, also known as UBE2I), and multiple SUMO E3 ligases. SUMO proteins themselves and SUMO E1, E2, and some, but not all, E3 enzymes are homologous to their counterparts in the ubiquitylation pathway.

The SUMOylation cascade begins with proteolytic maturation of a SUMO protein, which reveals the C-terminal -GG motif. The E1 enzyme activates mature SUMO in an ATP-dependent manner, producing an AMP∼SUMO intermediate (where “∼” represents a labile covalent bond). The AMP moiety is then replaced by a thioester bond to the active-site cysteine (C173) of the SAE2 subunit, resulting in SAE2∼SUMO. From there, SUMO is transferred onto C93 within the E2 UBC9, generating the UBC9∼SUMO thioester. The final step involves a direct transfer of SUMO from UBC9∼SUMO onto a lysine residue of a substrate, producing a substrate−SUMO conjugate, where “−“ denotes a chemically stable isopeptide bond between the substrate lysine amino group on the substrate and the C terminus of mature SUMO.

UBC9 plays an active role in substrate and site selection by interacting with certain sequence motifs in substrates, especially Ψ-K-X-E/D (where Ψ is a hydrophobic and X any amino acid residue) (1, 9). However, SUMOylation sites that do not conform to this consensus are known, including K14 in human UBC9 itself (auto-SUMOylation) and an equivalent residue in UBE2K (10, 11). UBC9 not only recognizes but also activates the acceptor site, notably through D127, which has been proposed to promote deprotonation of the substrate lysine residue to increase its nucleophilicity (9, 12).

While E1 and E2 enzymes can be sufficient for SUMOylation, especially *in vitro*, SUMO E3 ligases can accelerate the transfer of SUMO from UBC9 onto the substrate by providing a structural scaffold that stabilizes UBC9∼SUMO, which otherwise remains flexible and lowly reactive. In the presence of an E3 ligase, UBC9∼SUMO adopts a specific active conformation, termed the ‘closed conformation’ (13, 14, 15, 16, 17). The stabilization of the closed E2∼modifier conformation is a conserved mechanism of E2 activation by E3 ligases that has also been demonstrated for canonical RING-containing ubiquitin-specific E3s (18, 19, 20, 21, 22). In addition, SUMO E3 ligases can help recruit and orient the substrate (15, 16, 23).

The initial SUMO modification installed on a substrate can be extended to a polySUMO chain in a process that involves the ligation of a succeeding SUMO molecule to a lysine on a preceding SUMO. Chain formation depends on a noncovalent interaction between SUMO and the “backside” of UBC9 (24, 25, 26) and possibly on contact between two different UBC9 molecules (24, 26, 27); this process can be accelerated by an E3 ligase (15).

To gain a structural understanding of the SUMOylation reaction, structures of protein complexes including UBC9, SUMO molecule(s), an E3 ligase, and a substrate (or at least some of these elements) are required. SUMO should ideally be covalently linked either to UBC9 (reflecting the situation prior to the transfer) or to the substrate (reflecting the post-reaction state), or to both. Since the UBC9∼SUMO thioester is chemically unstable on the order of hours (28)—and more so in the presence of an E3 ligase and/or a good substrate—crystallographic studies have initially focused on the post-reaction complex with the C-terminal domain of RANGAP1 (RANGAP1^CTD^) as a model SUMOylation substrate. RANGAP1 is an unusual UBC9 substrate that evolved to strongly interact, particularly in its SUMOylated form, with UBC9 as part of a larger assembly (29). The human version of the UBC9:RANGAP1^CTD^−SUMO complex (containing either SUMO1 or SUMO2) has been co-crystallized with fragments of human SUMO E3 ligases RANBP2 (13, 30) or ZNF451 (14), showing how these structurally different E3s stabilize SUMO relative to UBC9 in a way that would correspond to the closed UBC9∼SUMO conformation.

An alternative strategy involves imitating the pre-reaction state using a chemically stable mimetic of the E2∼SUMO thioester. Streich Jr and Lima applied a mutagenesis-based biochemical approach to creating a mimetic composed of E2 and SUMO proteins from *S. cerevisiae*, Ubc9, and Smt3 (16, 31). They tested two different mutants of Ubc9, with lysine substituted for either C93 itself or a residue that is proximal to it in space, A129. The purpose of these mutations was to encourage stable Smt3 attachment to the introduced lysine on Ubc9 when incubating these proteins with E1. Of the two mutations tried, it was the latter, A129K, that proved much more efficient at generating a stable Ubc9–Smt3 linkage. The site of Smt3 ligation on Ubc9 was close in space to C93, thus structurally mimicking the thioester while leaving C93 itself untouched. This allowed additional covalent cysteine-to-cysteine crosslinking of a substrate protein (with the acceptor lysine mutated to a cysteine) *via* a specific homobifunctional crosslinker. The tripartite substrate–Ubc9–Smt3 complex was subsequently co-crystallized with a fragment of the yeast Siz1 E3 ligase. In the resultant structure, SUMO was again oriented by the E3 in a way that corresponds to the closed E2∼SUMO conformation (16). Streich Jr and Lima’s strategy has later been successfully applied by the Reverter group, this time without concomitant substrate crosslinking, to solve a structure of the Ubc9∼Smt3 mimetic bound to the yeast E3 ligase Nse2, which once more revealed a closed conformation stabilized by an E3 (17).

While more sophisticated, chemistry-assisted strategies for generating a human E2∼SUMO mimetic have recently been proposed by the Bode (32) or Melchior and Mootz (33) groups, the mutagenesis-based approach developed by Streich Jr and Lima remains highly attractive due to its ease of implementation and high yield. Here, we successfully applied it to generate a stable mimetic of the human UBC9∼SUMO1 thioester. On the way to this goal, we determined the factors that contribute to efficient UBC9 auto-SUMOylation on the introduced lysine. Finally, we solved a crystal structure of the UBC9–SUMO molecule, which reveals an open conformation of the mimetic and formation of noncovalent polymers in the crystal.

## Results

### Reconstitution of the SUMOylation reaction with and without a substrate

**Figure 1:**
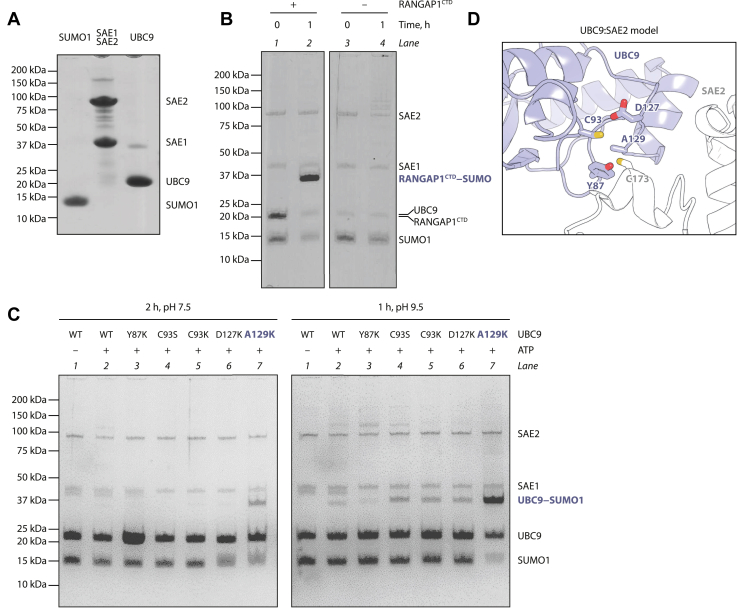
**Reconstitution of UBC9 auto-SUMOylation.***A*, gel migration control of purified tagged human SUMO1, SAE1:SAE2, and UBC9. *B*, SUMOylation reaction with ATP in the presence or absence of the model RANGAP1^CTD^ substrate. *C*, auto-SUMOylation of WT or mutant UBC9 under two sets of conditions. *D*, homology model of a fragment of the UBC9:SAE2 interaction, obtained by alignments of fragments of PDBs 1U9B and 1Y8Q on 5KNL. Side chains of cysteine SUMO donor residues from SAE2 and UBC9 and residues mutated in panel C are shown as *sticks*.

We began by generating vectors for bacterial production of N-terminally His_6_-tagged variants of the human SAE1:SAE2 heterodimer (bicistronic plasmid encoding a tagged SAE2 and untagged SAE1), UBC9, and SUMO1. For SUMO1, we trimmed the expressed region to residues 18 to 97, which correspond to a C-terminally processed form of SUMO1 with an additional deletion of an N-terminal flexible region that could hinder crystallization. The proteins were produced, purified (Fig. 1*A*), and used to reconstitute the SUMOylation reaction *in vitro*.

Unless otherwise stated, we carried out all SUMOylation reactions at 37 °C and treated all samples with dithiothreitol (DTT) to cleave thioester intermediates prior to SDS-PAGE (34, 35).

The activity of the purified proteins was first validated using RANGAP1^CTD^ as a substrate. RANGAP1^CTD^ is known to be efficiently SUMOylated in the absence of an E3 ligase due to its strong noncovalent interaction with UBC9 (9). Upon 1-h incubation with SAE1:SAE2, UBC9, SUMO1, and ATP at pH 7.5, the RANGAP1^CTD^ band was replaced by a single higher band consistent with mono-SUMOylated RANGAP1^CTD^ (Fig. 1*B*, compare lane 2 to 1).

In the absence of RANGAP1^CTD^, incubation under equivalent conditions led only to traces of SAE1, SAE2, and UBC9 SUMOylation, consistent with previous reports (24, 35, 36) (Fig. 1*B*, compare lane 4 to 3).

### Lysine substitution for A129 leads to efficient mono-SUMOylation of human UBC9

We subsequently repeated the SUMOylation reaction without RANGAP1^CTD^, this time increasing the amount of UBC9 to better visualize UBC9 auto-SUMOylation.

We varied the pH, testing two sets of conditions in parallel: a 2-h incubation at pH 7.5 (HEPES buffer) or a 1-h incubation at pH 9.5 (CHES buffer). Although in this and other experiments we used different buffering molecules for low and high pH, we believe that the difference in the result is attributable to the difference in pH rather than the chemical composition of the buffer. As SUMOylation proceeds through a nucleophilic attack by the acceptor residue, increasing the pH should stimulate the reaction by increasing the acceptor's nucleophilicity through deprotonation (12). Indeed, we observed the formation of a weak band consistent with mono-SUMOylated UBC9 at pH 9.5 but not (or only a very faint one) at pH 7.5 (Fig. 1*C*, compare lanes 2 at pH 7.5 and 9.5 with each other). This band likely corresponds to UBC9 auto-SUMOylation on various lysine sites, as previously reported (24, 35). The apparent auto-SUMOylation of WT UBC9 was not produced in control reactions without ATP (lanes 1).

In addition to the wild-type (WT) UBC9, we tested mutant UBC9 variants that feature a substitution of lysine or serine for C93 or a lysine substitution for residues proximal to C93 in space (Y87, D127, A129). We reasoned that placing a lysine or serine in the active site of UBC9 could promote efficient auto-SUMOylation through SUMO transfer onto the introduced acceptor either directly from C173 of SAE2 or from C93 within UBC9. An approximate model of the UBC9:SAE2 interaction, generated by superposing fragments of UBC9 (37) and SAE1:SAE2 (38) structures on a structure of a ubiquitin-specific E2:E1 complex (39), indicates the positions of the mutated residues in the vicinity of the cysteine SUMO donor sites on UBC9 and SAE2 (Fig. 1*D*).

Among the tested samples, the A129K mutant stood out for its efficient auto-SUMOylation (Fig. 1*C*, lanes 7 at pH 7.5 and 9.5), markedly stronger than that of the WT enzyme and other mutants. The auto-SUMOylation of UBC9 A129K was particularly efficient at pH 9.5, similar to what had been observed for the yeast Ubc9 A129K mutant (16).

Although the A129K mutation might have indirect effects on UBC9 auto-SUMOylation on other sites, the clear increase in UBC9 auto-SUMOylation in this mutant is most easily explained by SUMO ligation happening on the introduced lysine. Other analyzed mutants, such as C93K, C93S, or D127K, might also be auto-SUMOylated primarily on the introduced residue and could perhaps be used to create mimetics of UBC9∼SUMO, but it is less clear if this is the case and their auto-SUMOylation would need to be optimized to obtain a good yield.

### UBC9 A129K auto-SUMOylation depends on cysteine 93 and partially on D127

Focusing on the UBC9 A129K mutant, we attempted to gain insight into the mechanism of its efficient auto-SUMOylation.

**Figure 2:**
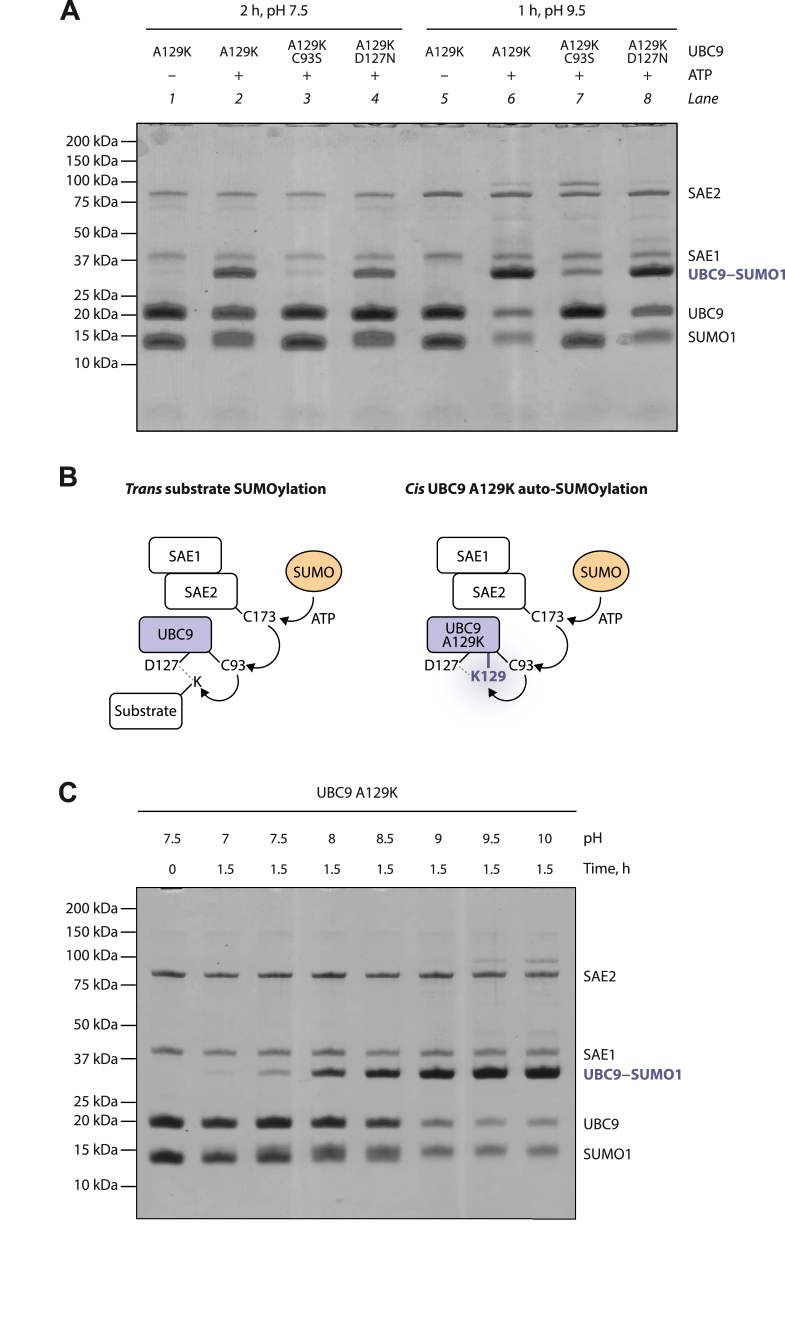
**Dependence of UBC9 A129K auto-SUMOylation on C93, D127, and pH.***A*, auto-SUMOylation of indicated UBC9 mutants under two sets of conditions. *B*, comparison of substrate SUMOylation in *trans* and UBC9 A129K auto-SUMOylation in *cis*. *C*, pH-dependent of UBC9 A129K auto-SUMOylation in the presence of ATP.

First, we investigated the path that SUMO takes to reach the acceptor residue in the mutant. It was previously observed that UBC9 (without the A129K mutation) can be auto-SUMOylated to a similar extent both in its WT and the C93A mutant form *in vitro*, suggesting that SUMO can be transferred onto certain lysine residues within UBC9 directly from SAE2 rather than through C93 of UBC9 (35). We asked if this is also the case for UBC9 A129K auto-SUMOylation by combining A129K and C93S mutations within UBC9 and comparing the apparent auto-SUMOylation of the resultant double mutant to that of UBC9 A129K at both pH 7.5 and pH 9.5. Mutating C93 dramatically impaired A129K auto-SUMOylation under both pH conditions (Fig. 2*A*, compare lanes 3 to 2 and 7 to 6). This suggests that SUMO is transferred onto the lysine in position 129 from C93, that is, following the same path as in the case of canonical SUMOylation of lysine residues in substrates (Fig. 2*B*). Therefore, the UBC9 A129K autoSUMOylation reaction, in addition to its practical use for generating a thioester mimetic, could also be considered a simplified model of substrate SUMOylation in which the “substrate” is constitutively present in the active site.

During canonical substrate SUMOylation, the acceptor lysine has been proposed to be activated by the UBC9 active site, particularly the residue D127 (12). To test if D127 plays a role in UBC9 A129K auto-SUMOylation, we combined A129K and D127N mutations and probed the auto-SUMOylation of the double mutant. We observed a small but reproducible decrease at pH 7.5, but only a slight or no decrease at pH 9.5 (Fig. 2*A*, compare lanes 4 to 2 and 8 to 6). This suggests that D127 may play a role in the enzymatic deprotonation of K129 at neutral pH.

### UBC9 A129K auto-SUMOylation is strongly pH-dependent

In the above experiments, UBC9 A129K auto-SUMOylation was more efficient at pH 9.5 than at pH 7.5. To investigate the pH dependence of the reaction more closely, we monitored the results of a 1.5-h-long reaction conducted at a range of pH values (from 7 to 10) (Fig. 2*C*). We observed a strong dependence of the amount of the generated product on pH, with a particularly steep increase in yield between pH 7.5 and 9.

This observation has two implications. First, it identifies optimal conditions for efficient generation of auto-SUMOylated UBC9 A129K. Second, it supports the notion that auto-SUMOylation of the A129K mutant in *cis* could serve as a simplified model of substrate SUMOylation (Fig. 2*B*), as for the latter similar pH dependence has been observed and attributed to the importance of lysine deprotonation (12).

### Large-scale production, purification, and crystallization of UBC9–SUMO1

**Figure 3:**
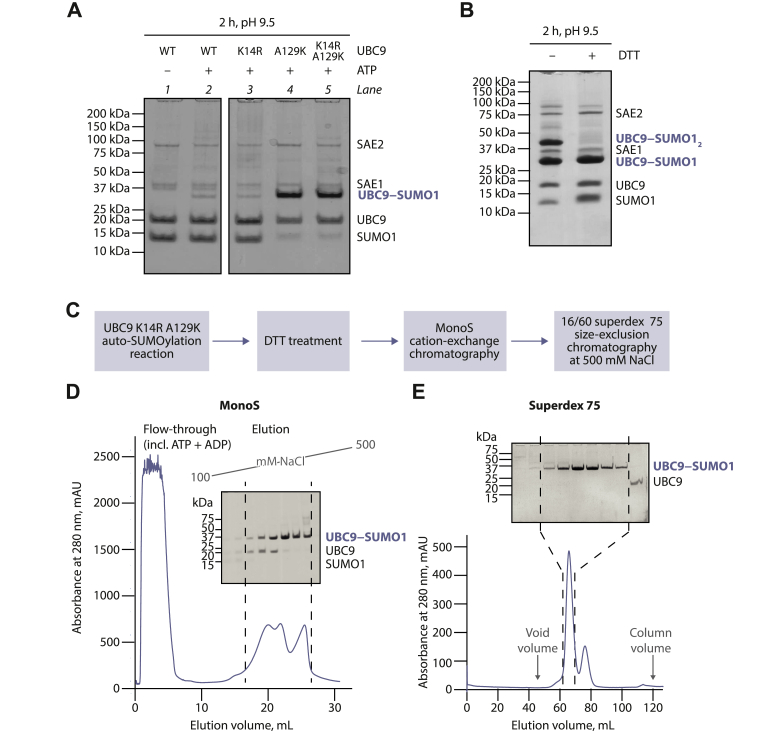
**Production and purification of UBC9–SUMO1.***A*, auto-SUMOylation of indicated UBC9 mutants. *B*, analysis of UBC9 K14R A129K auto-SUMOylation products treated or not with DTT prior to gel loading. *C*, mimetic production flowchart. *D* and *E*, cation-exchange (MonoS) and size-exclusion (Superdex 75) chromatography results, with gel analysis of indicated fraction ranges. In (*D*), a salt gradient is indicated.

The increased auto-SUMOylation observed for UBC9 A129K compared to the WT enzyme suggests that lysine 129 is modified much more efficiently than the native auto-SUMOylation sites and likely accounts for the majority of the auto-modification observed for the mutant. Nonetheless, to further discourage auto-SUMOylation on other sites, we decided—prior to large-scale production of the mimetic—to mutate also K14, the major auto-SUMOylation site previously reported for WT UBC9 (10). In a control experiment performed at pH 9.5, WT and K14R UBC9 were weakly auto-SUMOylated to a similar extent (Fig. 3*A*, lane 3 compared to 2). The sustained auto-SUMOylation of the K14R mutant is likely due to the presence of several minor SUMOylation sites (10, 35). Furthermore, when testing the effect of the K14R mutation in the A129K background, we saw that the single A129K and double K14R A129K mutants of UBC9 were strongly auto-SUMOylated to a similar extent (Fig. 3*A*, lane 5 compared to 4). Although these results do not show any effect of the K14R mutation on total auto-SUMOylation under our conditions, there might still be a “hidden” effect of K14R on the distribution of SUMO sites, which should favor K129 SUMOylation. We, therefore, chose the double K14R A129K mutant for large-scale mimetic production.

We next examined whether the auto-SUMOylation reaction modified UBC9 K14R A129K on C93 or possibly other cysteine residues through a thioester bond in addition to the observed lysine-linked mono-SUMOylation. Thioester-linked SUMOylation would not be visible in previous experiments due to the DTT treatment of all reaction products. Therefore, we now compared the products of auto-SUMOylation of UBC9 K14R A129K with or without DTT treatment. In the absence of DTT, we observed a higher band consistent with UBC9 being connected to two SUMOs, likely one *via* a lysine (primarily K129) and one *via* a cysteine (primarily C93) (Fig. 3*B*). Moreover, we presume that in samples without DTT, the band corresponding to UBC9 linked to a single SUMO includes some K- and some C-linked mono-SUMOylated UBC9. Upon DTT treatment, the higher band disappeared and bands for unconjugated UBC9 and SUMO became stronger, consistent with cleavage of thioester bonds. Based on this observation, we included a DTT treatment step in the protocol for mimetic production to eliminate thioester-linked SUMOylation and thus further increase the homogeneity of the product.

Following these preliminary tests, we carried out a large-scale auto-SUMOylation reaction using purified UBC9 K14R A129K, SAE1, SAE2, SUMO1, and ATP. We treated the product with DTT and purified it using a two-step protocol that included cation exchange and size-exclusion chromatography (Fig. 3, *C*–*E*). To minimize the likelihood of UBC9–SUMO and the remaining unmodified UBC9 strongly interacting with each other *via* the (predominantly polar) noncovalent backside binding site on UBC9, we performed the final size-exclusion chromatography step at high salt (500 mM NaCl) (Fig. 3*E*). The UBC9–SUMO1 molecule was obtained with good purity and its mass was verified using high-resolution mass spectrometry. The predominantly measured mass of 29147.1 Da is in good agreement with the theoretical mass of our UBC9 and SUMO1 constructs that additionally lack initiating methionine residues and are fused together *via* an isopeptide bond.

The purified UBC9–SUMO1 molecule was slow to concentrate in a spin concentrator and partially precipitated, suggesting limited solubility or an aggregation tendency, particularly when we tried to lower salt concentration. Crystallization trials performed with UBC9–SUMO1 alone were unsuccessful. As we were interested in the interaction between the UBC9–SUMO1 molecule and a putative SUMO E3 ligase TOPORS, we created a fusion of a short TOPORS-derived peptide and SUMO1 and added this fusion to our mimetic. This facilitated protein concentration and allowed us to obtain crystals, which, however, turned out to be built solely of UBC9–SUMO1. In light of the data presented below, we presume that UBC9–SUMO1 tends to self-associate into larger structures, which might lead to solubility issues. The added SUMO1 fusion might have facilitated concentration and crystallization by acting as a competitive inhibitor of self-association, although the added protein was not incorporated into the crystals.

### Crystal structure of the UBC9–SUMO1 molecule shows an open conformation

**Figure 4:**
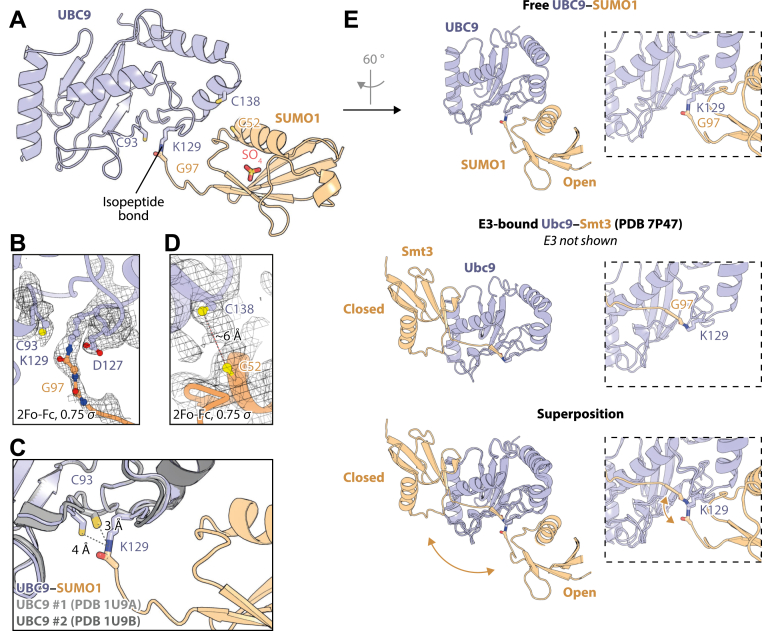
**Crystal structure of UBC9–SUMO1.***A*, overall view of UBC9–SUMO1. *B* and *D*, zoomed-in views of fragments of the structure with electron density. *C*, a fragment of a structural alignment of the mimetic structure with two different human UBC9 structures. *E*, comparison of our mimetic structure with the structure of the yeast E2∼SUMO mimetic in the closed conformation, with a structural alignment on the E2 at the *bottom*. Zoomed-in views of the active site are shown on the *right*.

The structure of the UBC9–SUMO1 molecule (*i.e.*, auto-SUMOylated UBC9 K14R A129K) was solved at a 2.85-Å resolution in the *P*2_1_2_1_2_1_ spacegroup (Table 1). There is a single UBC9–SUMO1 in the asymmetric unit, with both UBC9 and SUMO1 showing their characteristic fold (Fig. 4*A*).

**Table 1:** X-ray data collection and refinement statistics

Data collection statistics	
Radiation source	SOLEIL PROXIMA 1
Wavelength (Å)	0.97856
Spacegroup	*P*2_1_2_1_2_1_
Cell dimensions	
*a, b, c* (Å)	50.28, 53.58, 100.26
*α*, *β, γ* (°)	90.00, 90.00, 90.00
Resolution range (Å)	47.25–2.85 (3.00–2.85)
Total observations	60,913 (8784)
Unique reflections	6755 (949)
Completeness (%)	100.0 (100.0)
Multiplicity	9.0 (9.3)
*R*_*p.i.m.*_^a^ (%)	10.6 (50.6)
Average *I/*σ(*I*)	5.0 (1.3)
CC_1/2_ (%)	98.2 (68.2)
Resolution range (Å)	47.25–2.85
Number of reflections used	6715
*R*_work_^b^*/R*_free_^c^ (%)	22.52/25.79
Average B values (Å^2^)	
All atoms	65.45
UBC9 atoms	63.79
SUMO1 atoms	68.59
Sulfate atoms	104.69
Root mean square deviation from ideality	
Bond lengths (Å)	0.002
Bond angles (°)	0.504
Ramachandran analysis	
Favored regions/Allowed regions/Outliers (% of residues)	97.4/2.6/0.0
Number of atoms	
UBC9	1207
SUMO1	576
Sulfate	5
PDB code	8ODR

a $R_{p.i.m.}=\sum_{h}\sqrt{\frac{1}{n-1}}\sum_{i=1}^{n}\left|{I_{h,i}-\left\langle I\right\rangle }_{h}\right|/\sum_{h}\sum_{i}I_{h,i}$ where <*I>*_*h*_ is the mean intensity of the symmetry-equivalent reflections and n the redundancy.

b $R_{work}=\sum_{h}\left|\left|F_{o}\right|-\left|F_{c}\right|\right|/\sum_{h}\left|F_{o}\right|$, where *F*_*o*_ and *F*_*c*_ are the observed and calculated structure factor amplitudes, respectively, for reflection h.

c *R*_free_ is the *R* value for a subset of 10% of the reflection data, which were not included in the crystallographic refinement.

The active site residues of UBC9 are generally well resolved in the electron density map (Fig. 4*B*). The covalent link between lysine introduced in position 129 of UBC9 and G97 of SUMO1 is visible, too, but the C-terminal residues of SUMO1 exhibit high *B*-factors due to their flexibility. This is consistent with what has been observed in structures of other protein–SUMO conjugates (10, 40).

The N_*ζ*_ atom of K129 is only 4 Å from the S_*γ*_ atom of C93 (Fig. 4*C*), explaining why K129—which could move even closer when adopting a different rotamer—efficiently accepts SUMO1 from C93. Comparing our structure to those of free WT human UBC9 that were previously solved in two different space groups shows that C93 is shifted by about 1 Å compared to its position in free WT UBC9. This means that, in our conjugate, SUMO1 is linked 3 Å away from its attachment site in a real UBC9∼SUMO1 thioester conjugate, suggesting that our conjugate should serve as a suitable structural mimetic.

C52 of SUMO1 and C138 of UBC9 (both from the same UBC9-SUMO1 molecule) are relatively close to each other in space, with a distance of ∼6 Å between their sulfur atoms (Fig. 4*D*). There is also unassigned electron density between the two cysteines, which we hypothesize could correspond to a DTT molecule that crosslinked these two cysteine residues in a portion of molecules in the crystal. Of note, in a previously reported case of a DTT-mediated crosslink of two cysteines in another protein, the distance between sulfur atoms was also ∼6 Å (41).

We also observed an apparent sulfate ion, likely originating from the crystallization solution, near SUMO1 (Fig. 4*A*).

We next structurally aligned, on E2 molecules, our UBC9–SUMO1 structure with that of an equivalent mimetic of the yeast Ubc9∼Smt3 thioester (17) (Fig. 4*E*). The Ubc9∼Smt3 mimetic, which was also obtained using the A129K mutation, was co-crystallized with an active fragment of the SUMO E3 ligase Nse2, which stabilizes it in the closed conformation. The comparison with our structure shows that even though most of the K129 sidechain in both structures is superimposable, SUMO molecules are positioned in completely different locations relative to the E2, reflecting two radically different conformations of E2–SUMO. Our mimetic does not adopt the closed conformation observed for activated E2∼modifier molecules, instead featuring an open conformation. This is consistent with the idea that, in the absence of an E3 ligase, the thioester (or, in this case, its mimetic) samples various conformations. The actual conformation adopted in the crystal is likely affected by crystal packing.

### UBC9–SUMO1 molecules form noncovalent chains in the crystal

**Figure 5:**
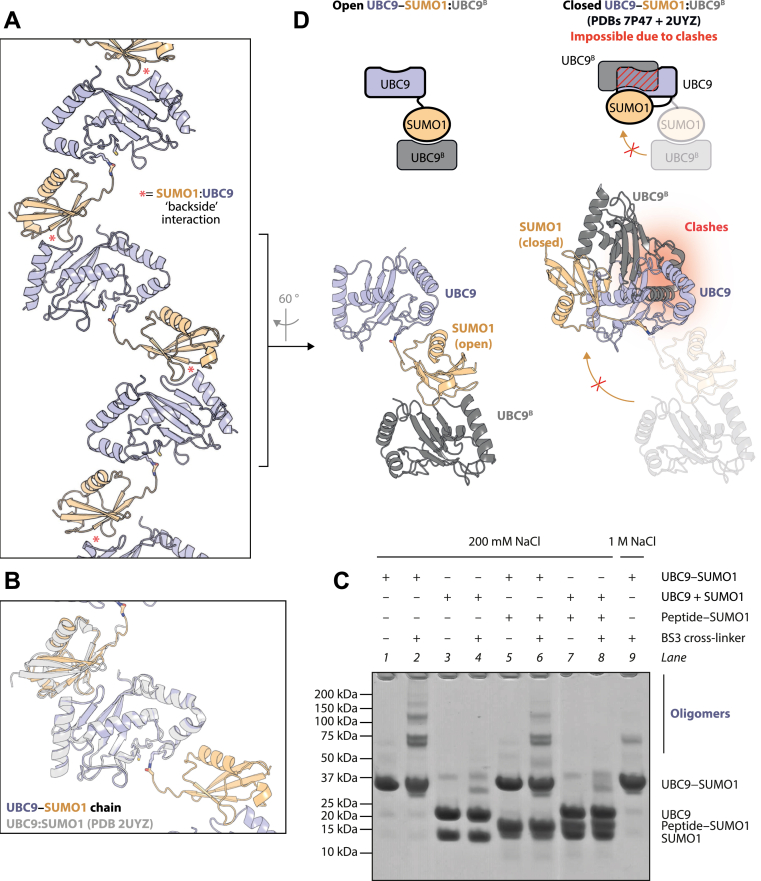
**Noncovalent oligomerization of UBC9–SUMO1.***A*, UBC9–SUMO1 chains formed in the crystal *via* noncovalent SUMO1:UBC9 interactions. *B*, structural alignment of a SUMO1:UBC9 interaction as seen in our mimetic structure and the previous UBC9:SUMO1 co-crystal structure. *C*, SDS-PAGE analysis of products of BS3-mediated cross-linking and control reactions without BS3. UBC9 + SUMO1 indicates an equimolar mixture of free UBC9 and SUMO1, as opposed to the covalently linked UBC9–SUMO1. *D*, a fragment of the chain, with a UBC9–SUMO1 molecule bound *via* SUMO to another UBC9 molecule (UBC9^B^). *Left*, this complex is in an open conformation as seen in our structure. *Right*, this complex modeled in a closed conformation based on PDBs 7P47 and 2UYZ shows steric incompatibility between two UBC9 molecules.

When scrutinizing crystal contacts, we observed that UBC9–SUMO1 molecules form a chain: UBC9–SUMO1:UBC9–SUMO1:UBC9–SUMO1… (where “–“ denotes a covalent and “:” a noncovalent interaction) (Fig. 5*A*). In this chain, SUMO1 from one covalent conjugate is noncovalently bound to UBC9 from a neighboring mimetic molecule through the well-characterized backside interaction (24, 25, 42, 43, 44) (Fig. 5*B*).

Since the non-covalent interaction between SUMO and UBC9 is known to have a relatively low dissociation constant, *K*_*D*_, of ∼100 nM (24), we predict that, in solution, the pure UBC9–SUMO1 molecule present at a sufficiently high concentration should oligomerize in a similar fashion to that observed in the crystal, with the number of protomers in a complex depending on protein concentration. These putative oligomers appear to be destabilized at high salt used during preparative size-exclusion chromatography. We were not able to test the oligomerization state at lower salt using size-exclusion chromatography due to the mimetic’s limited solubility and a tendency to stick to chromatography columns, properties that might be related to noncovalent oligomerization. Instead, we conducted a cross-linking experiment using a water-soluble protein cross-linker called bis(sulfosuccinimidyl)suberate (BS3) and protein samples at a low concentration of 10 μM. By covalently linking protein amino groups that are in close proximity, BS3 captures noncovalent protein oligomeric states present in the solution and allows their visualization through SDS-PAGE. When subjected to BS3-mediated cross-linking at 200 mM NaCl, UBC–SUMO1 produced a ladder of high-molecular-weight oligomeric states (Fig. 5*C*, compare lane 2 to 1). In a control reaction with an equimolar mixture of free UBC9 and SUMO1, no similar ladder was observed (compare lane 4 to 3). The addition of SUMO1 fused with a short TOPORS-derived peptide (the same one as present during crystallization) to UBC–SUMO1 resulted in a lower intensity of high-molecular-weight species produced upon cross-linking (compare lane 6 to lane 2), This suggests a reduction in noncovalent chain formation by the mimetic upon mixing with this SUMO1 fusion, consistent with our idea that it facilitated crystallization by buffering chain formation during crystal growth. Finally, increasing the NaCl concentration to 1 M abolished the formation of large cross-linked species (compare lane 9 to lane 2), in line with a decrease in oligomerization at higher ionic strength, a property that we leveraged during mimetic purification.

Our structure shows that the contact between two neighboring UBC9–SUMO molecules in the chain is limited to the SUMO:UBC9 interface. This means that there is no obvious structural reason why, in a complex cellular milieu, UBC9∼SUMO thioesters should preferentially interact with each other rather than with UBC9 and SUMO that are either free or, in the case of SUMO, conjugated to other proteins. Therefore, while we would expect purified UBC9∼SUMO to form homooligomers *in vitro*, we do not expect it to be a prevalent form in cells, except perhaps in areas with a high local concentration of the thioester. Instead, we presume that the self-association observed in our crystal structure represents just one possible link within a complex network comprising not only UBC9∼SUMO but also free UBC9 or SUMO and SUMOylated proteins, to mention just the interactions mediated by the high-affinity SUMO:UBC9 interface. Of note, a previously-reported crystal structure of UBC9 SUMOylated on K14 also reveals noncovalent chains mediated by SUMO:UBC9 interactions (10). Again, we would presume that, in cells, K14-SUMOylated UBC9 is likely to enter into a network with other components rather than preferentially homo-oligomerizing.

**Figure 6:**
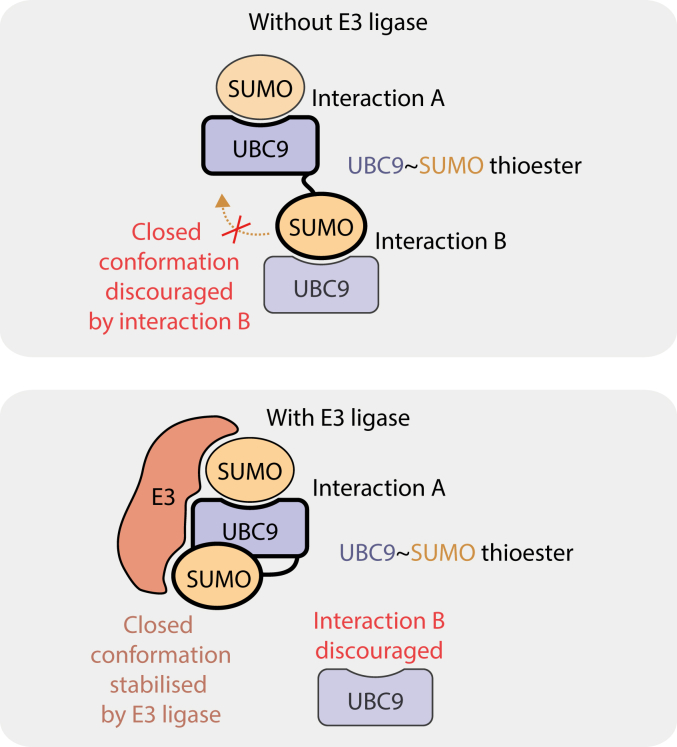
**A model of the predicted impact of SUMO E3 ligase binding on interactions of UBC9∼SUMO with auxiliary SUMO or UBC9 molecules**.

We next considered the possible effects on UBC9∼SUMO of being part of a larger homo- or heterotypic assembly mediated by UBC9:SUMO contacts. The UBC9∼SUMO thioester can bind *via* its UBC9 part to a SUMO molecule (defined here as interaction A) or *via* its SUMO part to a UBC9 molecule (interaction B). Interaction A is known to be compatible with the adoption of the closed conformation by the UBC9∼SUMO thioester and to even encourage the binding of some SUMO E3 ligases (14, 15, 16). It is also implicated in SUMO chain synthesis (15, 24, 25, 26). In contrast, the effects of interaction B have not been closely considered in the literature. To gain insights into its possible effects, we asked if such an interaction would be compatible with UBC9∼SUMO adopting the closed conformation (Fig. 5*D*). We therefore superposed the yeast Ubc9∼Smt3 mimetic captured in the closed conformation (17) and a noncovalent SUMO:UBC9 complex (24). The structural alignment of these two structures on SUMO clearly shows that the closed conformation of the UBC9∼SUMO thioester is not sterically compatible with SUMO interacting with the backside of another UBC9 molecule (Fig. 5*D*, right). This implies that interaction B should inhibit the adoption of the closed conformation by UBC9∼SUMO and vice versa (Fig. 6, top). An E3 ligase, in order to stabilize the closed conformation, would have to break interaction B (Fig. 6, bottom), although known E3s and UBC9 do not directly compete for binding to the same SUMO surface.

In summary, the contacts formed by the UBC9–SUMO molecule in the crystal highlight the ability of the thioester to engage in interactions *via* both its UBC9 and SUMO parts, possibly as part of a larger interaction network. These interactions are likely to impact the conformational landscape and possibly other properties of UBC9∼SUMO.

## Discussion

In this study, we attempted to create a stable mimetic of the human UBC9∼SUMO thioester by replacing one of the UBC9 active-site residues with a lysine residue capable of becoming stably SUMOylated. While more sophisticated approaches that rely on chemical protein synthesis (32) or non-native amino-acid incorporation and click chemistry (33) have been reported, a mutagenesis-based strategy is easier to implement in a biochemistry or structural biology laboratory and better suited for obtaining sufficient material for structural analysis.

The standard mutagenesis-based approach for creating mimetics of E2∼ubiquitin involves mutating the active-site cysteine of an E2 enzyme to lysine (19, 20, 21, 45, 46, 47). However, Streich Jr and Lima observed that, in the case of the yeast SUMO-specific E2 Ubc9, substituting lysine for a residue that is proximal to C93 in space, A129, provides a more efficient way of creating a mimetic (16, 31). Building on their findings and transposing them to the human UBC9 enzyme, we tested lysine substitutions in several positions in the active site of UBC9, observing that the A129K mutant was by far the most efficiently auto-SUMOylated. We presume that to induce strong auto-SUMOylation, a mutation has to place a SUMO acceptor in a correct location with respect to the donor cysteine residue on either UBC9 or SAE2 and possibly with respect to UBC9’s catalytic residues that could orient and activate it for the reaction. Additionally, the mutation has to avoid any negative effects on the UBC9:SAE2 interaction and—if UBC9 is catalytically implicated—on the integrity of the UBC9 active site. It appears that these criteria are best fulfilled by A129K. Auto-SUMOylated UBC9 A129K is not only efficiently produced, but should also be a good structural mimetic of the native UBC9∼SUMO thioester, as suggested by our crystal structure in which SUMO is attached just 3 Å from its native linkage. While we used SUMO1 in our study, we presume that a similar approach can be used for SUMO2/3 and that the insights described below are at least partially generalizable to all human SUMO paralogues.

Our biochemical data imply that SUMO is transferred onto the lysine introduced in position 129 from C93 of UBC9, that is, in the same way as it is onto a substrate lysine. Thus, in addition to its practical application as a means to mimetic generation, the UBC9 A129K auto-SUMOylation reaction could be used as a simplified model of substrate SUMOylation, from which the substrate recruitment step is eliminated because the “substrate” is constitutively present in the active site. In this sense, UBC9 A129K auto-SUMOylation could provide an alternative to the UBC9 version of the commonly used E2 discharge assay, where a preformed E2∼modifier thioester is incubated with a high concentration of free amino acid lysine (48). We show above that UBC9 A129K auto-SUMOylation is pH-dependent in a similar manner to substrate SUMOylation (12) and that it is partially dependent on D127 of UBC9, which presumably helps to position and deprotonate K129. Previously, the D127A mutations were shown to have an apparently stronger effect on substrate SUMOylation at neutral pH than the now-observed effect of the D127N mutation on UBC9 A129K auto-SUMOylation (9, 12), but this might be largely due to the difference in the exact mutation used. The effect of the D-to-N substitution more directly indicates a possible catalytic-base aspect to D127’s function. Of note, while D127 is conserved in animal UBC9 proteins, it is replaced by a serine in yeast orthologues, in line with this residue being partially dispensable for the reaction.

Our structure of the mimetic reveals an open conformation. This was expected considering that the structure captures the mimetic in the absence of an E3 ligase, which promotes the closed conformation. We predict that, in solution, there is no single open conformation; instead, UBC9∼SUMO likely samples a spectrum or ensemble of states, of which the one visualized in our crystal structure may be the dominant one or may have been favored during crystallization for other reasons. It is conceivable, but unlikely, that this conformation became stabilized in solution by the apparent DTT-mediated cysteine-cysteine crosslink discussed below. Instead, we believe that the protein crystallized in the observed conformation first, and the apparent DTT adduct occurred in the crystal owing to the two cysteines being optimally spaced for the reaction.

The two cysteines that appear to have been partially crosslinked are C138 of UBC9 and C52 of SUMO1. We believe that the spatial proximity of these two residues in the open mimetic conformation captured in our structure could hint at some functional connection between these two residues, *e.g.*, the formation of a regulatory disulfide bond under some conditions. A regulatory bond of this type could serve as one of the mechanisms for modulating UBC9's activity based on the cellular redox state, a phenomenon that has been reported in human cells (49, 50). Both of these cysteines are not found in yeast Ubc9 and Smt3, but they are present in animal UBC9 and SUMO proteins (not only in SUMO1 (C52) but also SUMO2/3 (C48/47)). Their functional relevance is currently unclear beyond the importance of C52 for SUMO’s thermal stability, particularly in SUMO1 (51, 52). New insights into these residues are needed, especially as cysteines can play particularly important roles in ubiquitylation-like systems.

Noteworthy, in the crystal, the UBC9–SUMO molecule is organized into “infinite” chains through a noncovalent SUMO:UBC9 interaction reported before (24, 25). E2∼ubiquitin mimetics also tend to make chains *via* an equivalent interaction in crystals, and it has been proposed that these chains reflect an oligomerization propensity of E2∼ubiquitin thioesters in solution (53). We believe that this could also be the case for UBC9∼SUMO, in which case oligomerization should be more pronounced owing to a considerably lower dissociation constant (in the medium nanomolar range) of the SUMO:UBC9 interaction compared to that between ubiquitin and its cognate E2s (in the medium micromolar range) (7, 14, 16, 24, 54). Therefore, oligomers should be considered during *in vitro* experiments performed with a purified thioester or its mimetic, and their existence can explain some previous observations. Noteworthy, the mimetic of yeast Ubc9∼Smt3 has been shown to elute early from a size-exclusion chromatography column and, while the authors did not comment on this phenomenon, it would be compatible with oligomerization (see Extended Data Fig. 1*C* in (16) and Fig. 5*D* in (17)). Furthermore, our structural alignment suggests that E3 ligase binding to UBC9∼SUMO is mutually exclusive with oligomerization *via* the SUMO part of UBC9∼SUMO. This again is consistent with the mentioned size-exclusion chromatography analyses of the yeast Ubc9–Smt3 molecule, where it appears to form smaller assemblies upon adding an E3 ligase.

In a complex milieu in which UBC9∼SUMO coexists with free UBC9, free and substrate-linked SUMO proteins, and other components, the proposed UBC9∼SUMO homo-oligomers will likely give way to more heterogeneous assemblies. However, even in such complexes, a given UBC9∼SUMO molecule would be expected to be able to form interactions similar to those in the crystal, that is, *via* the UBC9 part with a SUMO molecule (what we termed interaction A) and *via* SUMO with a UBC9 molecule (interaction B). These interactions might exert physiologically-relevant regulatory effects on the thioester, for example, by encouraging (interaction A) or competing with (interaction B) E3 ligase binding. The ratio of SUMO and UBC9 in a system would likely determine the extent to which interactions A and B occur. In a context where SUMO is in large excess over UBC9, UBC9∼SUMO would rarely make interaction B because UBC9 molecules would be saturated with SUMO. That is likely the case for an average cell, but the situation might change depending on a cell type, conditions, or subcellular location.

We observed that, perhaps due to oligomerization, the UBC9–SUMO1 molecule has solubility issues at low ionic strength. However, the molecule is well-behaved in adjusted conditions and we predict that it will be possible to use it to reconstitute complexes with SUMO E3 ligase fragments, which, once formed, should have different solubility properties. A substrate can probably be covalently crosslinked to the free C93 of UBC9–SUMO using a strategy also proposed by Streich Jr and Lima (16, 31), although this might require prior mutation of exposed cysteine residues including C138 of UBC9 and C52 of SUMO. Ultimately, this would allow generating covalently-stabilized ternary complexes for structural analysis.

Overall, this study serves as a possible first step to better characterizing human SUMOylation complexes, while already offering some new insights into SUMOylation.

## Experimental procedures

### Plasmids and mutagenesis

Codon-optimized (for *Escherichia coli*) DNA sequence encoding a fragment of the human SUMO1 protein (residues 18–97 of the UniProt sequence P63165|SUMO1_HUMAN) was synthesized by GenScript and cloned into the pET-28a vector. The part of the vector sequence encoding the thrombin cleavage site was subsequently deleted, resulting in SUMO1 being produced with an N-terminal tag MGSSHHHHHH.

The DNA sequence encoding human UBC9/UBE2I (UniProt P63279|UBC9_HUMAN) without the initial methionine was cloned from a commercial CpUC19 vector (Sino Biological cat. no. G08N024M70) into a pET28a vector in a way that confers an N-terminal tag MGHHHHHH. The protein sequence with a tag is shown below, with residues mutated in this study highlighted in bold:

MGHHHHHHSGIALSRLA QER**K**AWRKDH PFGFVAVPTK NPDGTMNLMN WECAIPGKKG TPWEGGLFKL RMLFKDDYPS SPPKCKFEPP LFHPNV**Y**PSG TV**C**LSILEED KDWRPAITIK QILLGIQELL NEPNIQ**D**P**A**Q AEAYTIYCQN RVEYEKRVRA QAKKFAPS

The expression vector for the full-length human SAE1:SAE2 heterodimer (UniProt sequences Q9UBE0|SAE1_HUMAN and Q9UBT2|SAE2_HUMAN) was obtained by deleting the sequence corresponding to SUMO1 and UBC9 from an Addgene-deposited pSUMO1 vector (plasmid no. 52258) (55). The resultant vector encodes MGSSHHHHHH-tagged SAE2 and untagged SAE1.

Codon-optimized (for *E. coli*) DNA sequence coding for the C-terminal domain of RANGAP1 (RANGAP1^CTD^, residues 419–587) was synthesized by GenScript and cloned into the pET-28a vector conferring an N-terminal MGSSHHHHHHSSGLVPRGSHMSN tag.

A plasmid for the production of a short TOPORS fragment (residues 468–501 of UniProt sequence Q9NS56|TOPRS_HUMAN) followed by residues 18 to 97 of SUMO1 was created by insertion of a codon-optimized *TOPORS* fragment sequence into the above-mentioned *SUMO1* pET-28a plasmid, between sequences encoding the tag and SUMO1.

All mutations were produced using either a standard mutagenesis PCR followed by DpnI treatment or the Q5 Site-Directed Mutagenesis Kit (New England BioLabs, cat. no. E0554S).

### Protein production and purification

All proteins were expressed in the *E. coli* strain BL21 (DE3) cultured in lysogeny broth (LB) medium supplemented with 50 μg/ml of kanamycin (for all pET-28a vectors) or 100 μg/ml of spectinomycin (for the modified pSUMO1 vector encoding SAE1:SAE2). Cultures were grown to an OD_600_ of ∼0.8 to 1.0 at 37 °C and induced with 0.5 mM isopropylthio-β-galactoside (IPTG). After 3 h of expression, the cells were harvested and frozen at −20 °C.

Defrosted cells were resuspended in HisTrap buffer (500 mM NaCl, 25 mM HEPES, pH 7.5, 20 mM imidazole, 5% glycerol, 1 mM DTT) supplemented with 0.5 mg/ml lysozyme. This cell suspension was incubated for 20 min at 25 °C, followed by a short sonication in a cold bath and centrifugation at 27,000*g* for 30 min. The supernatant was loaded onto a 5-ml HisTrap HP column (Cytiva, cat. no. 17-5248-01). After loading, the column was washed with HisTrap buffer supplemented with 30 mM imidazole prior to elution by an imidazole gradient (30–200 mM over 100 ml).

The proteins were further purified by ion exchange using a 5-ml HiTrap Q HP column (Cytiva, cat. no. 17-1154-01) for SUMO1, 5-ml HiTrap SP HP column (Cytiva, cat. no. 17-1152-01) for UBC9 and RANGAP1^CTD^, or 1-ml Mono Q 5/50 Gl column (Cytiva, cat. no. 17-5166-01) for SAE1:SAE2. The proteins eluted in the salt gradient around 200 mM, 300 mM, 250 mM, and 250 mM NaCl, for SUMO1, UBC9, RANGAP1^CTD^, and SAE1:SAE2, respectively. For all UBC9 variants, three peaks were observed in the elution gradient but only the third peak was retained due to its highest quality evaluated as per high-resolution mass spectrometry (HRMS) (correspondence with the expected mass and homogeneity).

In the last step, the proteins were purified by size-exclusion chromatography/gel filtration using a HiLoad Superdex 75 or 200 prep grade 16/600 column (Cytiva, cat. no. 28-9893-33 or 28-9893-35) equilibrated with 200 mM NaCl, 20 mM HEPES, pH 7.5, 5% of glycerol, and 1 mM DTT. The proteins were concentrated using Amicon 10 kDa concentrators (Merck Millipore cat. no. ACS501024) and stored at −80 °C. The concentration was determined by the absorbance at 280 nm. Protein identity was confirmed by HRMS analysis.

The TOPORS-SUMO1 fusion was produced and purified like SUMO1.

### SUMOylation reactions

Reactions were performed with different enzyme concentrations, as provided below, and at different pH and temperatures, as indicated in figures, in a total volume of 10 to 20 μl. The reaction buffer contained 150 mM NaCl, 5% glycerol, 5 mM MgCl_2_, and 20 mM of either HEPES (for pH 7–8.5) or CHES (for pH 9–10). Reaction mixtures in the experiment in Figure 1*B* included protein components at the following concentrations: 15 μM SUMO1, 1 μM UBC9, 1 μM SAE1:SAE2, and 15 μM RANGAP1^CTD^. In the experiments in Figure 1, Figure 2*A*, and 3A, the same concentrations were used except for UBC9, which was increased to 15 μM. In the experiments in Figures 2*C* and 3B, we used 30 μM SUMO1, 15 μM UBC9, 3 μM SAE1:SAE2. The reactions were triggered by adding 2 mM ATP and stopped by adding 5 μl of a gel-loading dye that contained 100 mM DTT. To see the complexes formed by the thioester link, a loading buffer without DTT was used and the samples were incubated or not with 100 mM DTT at 37 °C prior to adding the loading dye. Proteins were separated by SDS-PAGE and visualized with ReadyBlue (Sigma Aldrich, cat. no. RSB-1L).

### Generation of the UBC9–SUMO1 molecule

SUMO1 (180 nmol), UBC9 K14R A129K (120 nmol), and SAE1:SAE2 (5 nmol) were mixed in the buffer 150 mM NaCl, 5% glycerol, 5 mM MgCl_2_, 20 mM CHES, pH 9, in the final concentration of 300 μl. The reaction was incubated at 37 °C for 1 h from the moment of adding 2 mM ATP. At the end of the reaction, DTT (10 mM) was added to reduce the complexes formed *via* thioester bonds, and the reaction was further incubated at 37 °C for 1 h. The mixture was then diluted to adjust the NaCl concentration to 100 mM before injecting it onto a 1-ml Mono S 5/50 Gl column (Cytiva, cat. no. 17-5168-01) column equilibrated in 100 mM NaCl, 5% glycerol, 20 mM HEPES, pH7.5. The protein was eluted with a 100 to 500 mM NaCl gradient over 30 ml. The fractions of interest were pooled and concentrated for injection onto a Superdex 75 prep grade 16/600 column (Cytiva, cat. no. 28-9893-35) equilibrated with 500 mM NaCl, 20 mM HEPES, pH 7.5, 5% glycerol and 1 mM DTT.

### Crystallization of the UBC9–SUMO1 molecule

We performed crystallization using a protein mixture containing the UBC9–SUMO1 molecule at the final concentration of 170 μM and TOPORS-SUMO1 at 200 μM in 150 mM NaCl, 20 mM HEPES, pH 7.5. 5% glycerol, 11 mM DTT. Crystallization trials were performed at 20 °C using JCSG Plus, Morpheus, Wizard Classic 1 + 2, and Structure 1 + 2 screens from Molecular Dimensions (cat. no. MD1-37, MD1-46, MD15-W12-T, MD1-01, and MD1-02) *via* the sitting-drop vapor-diffusion method using a Mosquito liquid handling instrument (TTP LabTech). Crystals were detected after a few days in a Wizard Classics condition: 200 mM LiSO_4_, 100 mM Tris, pH 8.5, 30% PEG 4000.

### Data collection and structure determination

For X-ray data collection, a single crystal was transferred into a drop consisting of 3 μl of the well condition and 1 μl of 100% ethylene glycol. After a 5- to 10-s soak, the crystal was flash-cooled by rapidly plunging into liquid nitrogen. 100-K X-ray diffraction data were collected remotely on PROXIMA-1 beamline at the SOLEIL synchrotron. The diffraction data were processed using XDS (56) and AIMLESS (57). The crystal structure was determined by molecular replacement using Phaser (58) of the Phenix suite (59). The best solution was obtained using the two components of PDBid 2PE6 (UBC9 and SUMO1) as two independent molecules. The atomic model was refined using phenix.refine and manually improved using COOT (60). After final refinement rounds, *R*_free_ and *R*_work_ were calculated to be 22.52% and 25.79%, respectively. The data collection and refinement statistics are listed in Table 1. The model quality was validated by MolProbity (61) as implemented in Phenix. Molecular graphics images were produced using Pymol (62) or UCSF Chimera (63). As a note, no obvious electron density was observed for the TOPORS-SUMO1 molecule.

### Accession numbers

PDB: 8ODR.

### BS3 cross-linking

Protein samples (10 μM UBC9–SUMO1 or 10 μM UBC9 and 10 μM SUMO1) were prepared in 25 mM HEPES, pH 7.5 and either 200 or 1000 mM NaCl. 20 μM TOPORS-SUMO1 was added to indicated reactions. Following a 15-min incubation at room temperature, cross-linking was performed by adding 0.5 mM bis(sulfosuccinimidyl)suberate (BS3) and further incubating at room temperature for 15 min. The reactions were quenched with 100 mM Tris at pH 7.5 for 10 min at room temperature. Control reactions were treated in the same way but without adding BS3. Samples were precipitated with trichloroacetic acid (TCA) prior to SDS-PAGE.

## Data availability

Coordinates and structure factors have been deposited in the Protein Data Bank (PDB 8ODR).

## Conflict of interest

The authors declare that they have no known competing financial interests or personal relationships that could have appeared to influence the work reported in this paper.

## References

[1] Melchior F. (2000). SUMO—nonclassical ubiquitin. Annu. Rev. Cell Dev. Biol..

[2] Hay R.T. (2005). Sumo: a history of modification. Mol. Cell.

[3] Flotho A., Melchior F. (2013). Sumoylation: a regulatory protein modification in health and disease. Annu. Rev. Biochem..

[4] Celen A.B., Sahin U. (2020). Sumoylation on its 25th anniversary: mechanisms, pathology, and emerging concepts. FEBS J..

[5] Gareau J.R., Lima C.D. (2010). The SUMO pathway: emerging mechanisms that shape specificity, conjugation and recognition. Nat. Rev. Mol. Cell Biol..

[6] Pichler A., Fatouros C., Lee H., Eisenhardt N. (2017). SUMO conjugation – a mechanistic view. Biomol. Concepts.

[7] Cappadocia L., Lima C.D. (2018). Ubiquitin-like protein conjugation: structures, chemistry, and mechanism. Chem. Rev..

[8] Bouchard D., Wang W., Yang W.-C., He S., Garcia A., Matunis M.J. (2021). SUMO paralogue–specific functions revealed through systematic analysis of human knockout cell lines and gene expression data. Mol. Biol. Cell.

[9] Bernier-Villamor V., Sampson D.A., Matunis M.J., Lima C.D. (2002). Structural basis for E2-mediated SUMO conjugation revealed by a complex between ubiquitin-conjugating enzyme Ubc9 and RanGAP1. Cell.

[10] Knipscheer P., Flotho A., Klug H., Olsen J.V., van Dijk W.J., Fish A. (2008). Ubc9 sumoylation regulates SUMO target discrimination. Mol. Cell.

[11] Pichler A., Knipscheer P., Oberhofer E., van Dijk W.J., Körner R., Olsen J.V. (2005). SUMO modification of the ubiquitin-conjugating enzyme E2-25K. Nat. Struct. Mol. Biol..

[12] Yunus A.A., Lima C.D. (2006). Lysine activation and functional analysis of E2-mediated conjugation in the SUMO pathway. Nat. Struct. Mol. Biol..

[13] Reverter D., Lima C.D. (2005). Insights into E3 ligase activity revealed by a SUMO–RanGAP1–Ubc9–Nup358 complex. Nature.

[14] Cappadocia L., Pichler A., Lima C.D. (2015). Structural basis for catalytic activation by the human ZNF451 SUMO E3 ligase. Nat. Struct. Mol. Biol..

[15] Eisenhardt N., Chaugule V.K., Koidl S., Droescher M., Dogan E., Rettich J. (2015). A new vertebrate SUMO enzyme family reveals insights into SUMO-chain assembly. Nat. Struct. Mol. Biol..

[16] Streich F.C., Lima C.D. (2016). Capturing a substrate in an activated RING E3/E2–SUMO complex. Nature.

[17] Varejão N., Lascorz J., Codina-Fabra J., Bellí G., Borràs-Gas H., Torres-Rosell J. (2021). Structural basis for the E3 ligase activity enhancement of yeast Nse2 by SUMO-interacting motifs. Nat. Commun..

[18] Pruneda J.N., Littlefield P.J., Soss S.E., Nordquist K.A., Chazin W.J., Brzovic P.S. (2012). Structure of an E3:E2∼Ub complex reveals an allosteric mechanism shared among RING/U-box ligases. Mol. Cell.

[19] Plechanovová A., Jaffray E.G., Tatham M.H., Naismith J.H., Hay R.T. (2012). Structure of a RING E3 ligase and ubiquitin-loaded E2 primed for catalysis. Nature.

[20] Dou H., Buetow L., Sibbet G.J., Cameron K., Huang D.T. (2012). BIRC7–E2 ubiquitin conjugate structure reveals the mechanism of ubiquitin transfer by a RING dimer. Nat. Struct. Mol. Biol..

[21] Branigan E., Plechanovová A., Jaffray E.G., Naismith J.H., Hay R.T. (2015). Structural basis for the RING-catalyzed synthesis of K63-linked ubiquitin chains. Nat. Struct. Mol. Biol..

[22] Branigan E., Carlos Penedo J., Hay R.T. (2020). Ubiquitin transfer by a RING E3 ligase occurs from a closed E2∼ubiquitin conformation. Nat. Commun..

[23] Pichler A., Gast A., Seeler J.S., Dejean A., Melchior F. (2002). The nucleoporin RanBP2 has SUMO1 E3 ligase activity. Cell.

[24] Knipscheer P., van Dijk W.J., Olsen J.V., Mann M., Sixma T.K. (2007). Noncovalent interaction between Ubc9 and SUMO promotes SUMO chain formation. EMBO J..

[25] Capili A.D., Lima C.D. (2007). Structure and analysis of a complex between SUMO and Ubc9 illustrates features of a conserved E2-ubl interaction. J. Mol. Biol..

[26] Klug H., Xaver M., Chaugule V.K., Koidl S., Mittler G., Klein F. (2013). Ubc9 sumoylation controls SUMO chain formation and meiotic synapsis in Saccharomyces cerevisiae. Mol. Cell.

[27] Alontaga A.Y., Ambaye N.D., Li Y.-J., Vega R., Chen C.-H., Bzymek K.P. (2016). Observation of an E2 (Ubc9)-homodimer by crystallography. Data Brief.

[28] Song J., Wang J., Jozwiak A.A., Hu W., Swiderski P.M., Chen Y. (2009). Stability of thioester intermediates in ubiquitin-like modifications. Protein Sci..

[29] Werner A., Flotho A., Melchior F. (2012). The RanBP2/RanGAP1∗SUMO1/ubc9 complex is a multisubunit SUMO E3 ligase. Mol. Cell.

[30] Gareau J.R., Reverter D., Lima C.D. (2012). Determinants of small ubiquitin-like modifier 1 (SUMO1) protein specificity, E3 ligase, and SUMO-RanGAP1 binding activities of nucleoporin RanBP2∗. J. Biol. Chem..

[31] Streich F.C., Lima C.D. (2018). Strategies to trap enzyme-substrate complexes that mimic michaelis intermediates during E3-mediated ubiquitin-like protein ligation. Methods Mol. Biol..

[32] Zhang Y., Hirota T., Kuwata K., Oishi S., Gramani S.G., Bode J.W. (2019). Chemical synthesis of atomically tailored SUMO E2 conjugating enzymes for the formation of covalently linked SUMO–E2–E3 ligase ternary complexes. J. Am. Chem. Soc..

[33] Sommer S., Ritterhoff T., Melchior F., Mootz H.D. (2015). A stable chemical SUMO1–ubc9 conjugate specifically binds as a thioester mimic to the RanBP2–E3 ligase complex. ChemBioChem.

[34] Scheffner M., Nuber U., Huibregtse J.M. (1995). Protein ubiquitination involving an E1–E2–E3 enzyme ubiquitin thioester cascade. Nature.

[35] Subramaniam S., Mealer R.G., Sixt K.M., Barrow R.K., Usiello A., Snyder S.H. (2010). Rhes, a physiologic regulator of Sumoylation, enhances cross-sumoylation between the basic sumoylation enzymes E1 and Ubc9∗. J. Biol. Chem..

[36] Truong K., Lee T.D., Chen Y. (2012). Small ubiquitin-like modifier (SUMO) modification of E1 Cys domain inhibits E1 Cys domain enzymatic activity. J. Biol. Chem..

[37] Tong H., Hateboer G., Perrakis A., Bernards R., Sixma T.K. (1997). Crystal structure of murine/human Ubc9 provides insight into the variability of the ubiquitin-conjugating system∗. J. Biol. Chem..

[38] Lois L.M., Lima C.D. (2005). Structures of the SUMO E1 provide mechanistic insights into SUMO activation and E2 recruitment to E1. EMBO J..

[39] Olsen S.K., Lima C.D. (2013). Structure of a ubiquitin E1-E2 complex: insights to E1-E2 thioester transfer. Mol. Cell.

[40] Baba D., Maita N., Jee J.-G., Uchimura Y., Saitoh H., Sugasawa K. (2005). Crystal structure of thymine DNA glycosylase conjugated to SUMO-1. Nature.

[41] Valkov E., Stamp A., DiMaio F., Baker D., Verstak B., Roversi P. (2011). Crystal structure of Toll-like receptor adaptor MAL/TIRAP reveals the molecular basis for signal transduction and disease protection. Proc. Natl. Acad. Sci. U. S. A..

[42] Liu Q., Jin C., Liao X., Shen Z., Chen D.J., Chen Y. (1999). The binding interface between an E2 (UBC9) and a ubiquitin homologue (UBL1)∗. J. Biol. Chem..

[43] Bencsath K.P., Podgorski M.S., Pagala V.R., Slaughter C.A., Schulman B.A. (2002). Identification of a multifunctional binding site on Ubc9p required for Smt3p conjugation∗. J. Biol. Chem..

[44] Duda D.M., van Waardenburg R.C.A.M., Borg L.A., McGarity S., Nourse A., Waddell M.B. (2007). Structure of a SUMO-binding-motif mimic bound to Smt3p–ubc9p: conservation of a non-covalent ubiquitin-like protein–E2 complex as a platform for selective interactions within a SUMO pathway. J. Mol. Biol..

[45] Buetow L., Gabrielsen M., Anthony N.G., Dou H., Patel A., Aitkenhead H. (2015). Activation of a primed RING E3-E2-ubiquitin complex by non-covalent ubiquitin. Mol. Cell.

[46] Kumar P., Magala P., Geiger-Schuller K.R., Majumdar A., Tolman J.R., Wolberger C. (2015). Role of a non-canonical surface of Rad6 in ubiquitin conjugating activity. Nucleic Acids Res..

[47] Middleton A.J., Day C.L. (2015). The molecular basis of lysine 48 ubiquitin chain synthesis by Ube2K. Sci. Rep..

[48] Buetow L., Gabrielsen M., Huang D.T. (2018). Single-turnover RING/U-box E3-mediated lysine discharge assays. Methods Mol. Biol..

[49] Bossis G., Melchior F. (2006). Regulation of SUMOylation by reversible oxidation of SUMO conjugating enzymes. Mol. Cell.

[50] Stankovic-Valentin N., Drzewicka K., König C., Schiebel E., Melchior F. (2016). Redox regulation of SUMO enzymes is required for ATM activity and survival in oxidative stress. EMBO J..

[51] Drobecq H., Boll E., Sénéchal M., Desmet R., Saliou J.-M., Lacapère J.-J. (2016). A central cysteine residue is essential for the thermal stability and function of SUMO-1 protein and SUMO-1 peptide-protein conjugates. Bioconjug. Chem..

[52] Bouchenna J., Sénéchal M., Drobecq H., Stankovic-Valentin N., Vicogne J., Melnyk O. (2019). The role of the conserved SUMO-2/3 cysteine residue on domain structure investigated using protein chemical synthesis. Bioconjug. Chem..

[53] Page R.C., Pruneda J.N., Amick J., Klevit R.E., Misra S. (2012). Structural insights into the conformation and oligomerization of E2∼Ubiquitin conjugates. Biochemistry.

[54] Tatham M.H., Kim S., Yu B., Jaffray E., Song J., Zheng J. (2003). Role of an N-terminal site of Ubc9 in SUMO-1, -2, and -3 binding and conjugation. Biochemistry.

[55] Weber A.R., Schuermann D., Schär P. (2014). Versatile recombinant SUMOylation system for the production of SUMO-modified protein. PLoS One.

[56] Kabsch W. (2010). Xds. Acta Crystallogr. D Biol. Crystallogr..

[57] Evans P.R., Murshudov G.N. (2013). How good are my data and what is the resolution?. Acta Crystallogr. D Biol. Crystallogr..

[58] McCoy A.J. (2007). Solving structures of protein complexes by molecular replacement with Phaser. Acta Crystallogr. D Biol. Crystallogr..

[59] Adams P.D., Afonine P.V., Bunkóczi G., Chen V.B., Davis I.W., Echols N. (2010). Phenix: a comprehensive python-based system for macromolecular structure solution. Acta Crystallogr. D Biol. Crystallogr..

[60] Emsley P., Cowtan K. (2004). Coot: model-building tools for molecular graphics. Acta Crystallogr. D Biol. Crystallogr..

[61] Davis I.W., Leaver-Fay A., Chen V.B., Block J.N., Kapral G.J., Wang X. (2007). MolProbity: all-atom contacts and structure validation for proteins and nucleic acids. Nucleic Acids Res..

[62] DeLano W.L. (2002). Pymol: An open-source molecular graphics tool. CCP4 Newsl. Protein Crystallogr..

[63] Pettersen E.F., Goddard T.D., Huang C.C., Couch G.S., Greenblatt D.M., Meng E.C. (2004). UCSF Chimera—a visualization system for exploratory research and analysis. J. Comput. Chem..

